# Machine Learning Analysis of Maize Seedling Traits Under Drought Stress

**DOI:** 10.3390/biology14070787

**Published:** 2025-06-29

**Authors:** Lei Zhang, Fulai Zhang, Wentao Du, Mengting Hu, Ying Hao, Shuqi Ding, Huijuan Tian, Dan Zhang

**Affiliations:** 1 College of Agriculture, Tarim University, Alar 843300, China; 2005122037@163.com (L.Z.); zfl7127@163.com (F.Z.); t2273266657@163.com (W.D.); humtww@163.com (M.H.); hy18386110782@163.com (Y.H.); 1011219106@stumail.taru.edu.cn (S.D.); wmzthj@163.com (H.T.); 2 Key Laboratory of Genetic Improvement and Efficient Production for Specialty Crops in Arid Southern Xinjiang of Xinjiang Corps, College of Agronomy, Tarim University, Alar 843300, China

**Keywords:** maize, drought stress, seedling stage, machine learning

## Abstract

**Simple Summary:**

This study sought to understand how drought affects maize seedlings and identify traits linked to drought tolerance, aiming to aid in selecting and breeding more resilient maize varieties. Researchers grew 78 maize hybrids; normal watering was maintained until the development stage of three leaves and one heart, followed by a cessation of watering for 10 days; eight traits, like plant height, stem thickness, and chlorophyll content, were measured. Three machine learning methods were used to analyze the data and predict drought tolerance. The results showed that plant height, aboveground weight, and chlorophyll content are key traits for predicting drought tolerance. The XGBoost model was the most effective in classification tasks. This research provides valuable insights for developing maize varieties that can better withstand drought conditions, helping farmers and breeders improve crop resilience in the face of increasing drought risks due to climate change.

**Abstract:**

The increasing concentration of greenhouse gases is amplifying the global risk of drought on crop productivity. This study sought to investigate the effects of drought on the growth of maize (*Zea mays* L.) seedlings. A total of 78 maize hybrids were employed in this study to replicate drought conditions through the potting method. The maize seedlings were subjected to a 10-day period of water breakage following a standard watering cycle until they reached the third leaf collar (V3) stage. Parameters including plant height, stem diameter, chlorophyll content, and root number were assessed. The eight phenotypic traits include the fresh and dry weights of both the aboveground and underground parts. Three machine learning methods—random forest (RF), K-nearest neighbor (KNN), and extreme gradient boosting (XGBoost)—were employed to systematically analyze the relevant traits of maize seedlings’ drought tolerance and to assess their predictive performance in this regard. The findings indicated that plant height, aboveground weight, and chlorophyll content constituted the primary indices for phenotyping maize seedlings under drought conditions. The XGBoost model demonstrated optimal performance in the classification (AUC = 0.993) and regression (R^2^ = 0.863) tasks, establishing itself as the most effective prediction model. This study provides a foundation for the feasibility and reliability of screening drought-tolerant maize varieties and refining precision breeding strategies.

## 1. Introduction

Drought stress stands as a prominent abiotic constraint on global crop production, leading to substantial reductions in crop yield and quality [1]. Crop growth and development are greatly influenced by external environmental conditions [2,3]; as a global food crop, it is necessary to study the drought tolerance of maize under frequent adversity stresses, such as global warming and drought stress. Drought stress significantly inhibits maize plant height and root development, impeding the overall growth [4]; similarly, leaf number, leaf area index, maximum leaf area, and biomass accumulation also show a downward trend [5,6,7], leading to alterations in the accumulation of physiological and biochemical constituents, as well as changes in the protective enzyme activities [8], transpiration rate, and photosynthetic processes [9,10,11], thereby severely affecting its growth and development [12] and significantly diminishing yield and quality [13,14,15]. Drought tolerance in maize presents intricate characteristics governed by diverse regulatory mechanisms across different levels [16]. Drought stress at the seedling stage will affect the emergence rate of maize, which in turn will affect the number of ears harvested and yield. In order to study the negative effects of drought stress on maize production, it is necessary to further study the phenotypic response of maize seedlings under drought stress.

In maize drought tolerance research, traditional physiological indicator screening methods are inefficient and costly, while machine learning methods provide novel ideas for predicting crop drought tolerance by analyzing complex phenotypic data. Machine learning models possess efficient data processing capabilities, enabling the automatic identification and selection of key features through algorithms, thereby effectively reducing the subjectivity and errors associated with manual screening [17]. Moreover, these models can continuously optimize themselves based on new data, adapting to changes in different environments and varieties, which can enhance the accuracy and reliability of predictions. Through predictive modeling, researchers can rapidly screen for corn varieties with high drought tolerance potential, thereby minimizing unnecessary field trials and resource wastage [18]. This technology not only provides new ideas and technical means for traditional agricultural research but also propels the innovative development of agricultural science. In recent years, machine learning has gained significant traction and has been instrumental in crop genetic breeding endeavors [19]. For example, the combination of machine learning and crop modeling has demonstrated the capability to accurately predict maize yield [20,21]. Researchers used two maize hybrids with different drought phenotypic traits to assess their phenotypic changes under drought conditions; the maize hybrids of both genotypes had lower plant height and aboveground dry weight, smaller leaf area, and greater decreases in the underground dry weight under drought conditions compared to fully watered conditions [22]. Maize seedlings after two rounds of drought treatment had significantly lower plant height and leaf area and reduced biomass compared to the controls [23]. Moreover, machine learning methods have applications in many other aspects of maize, such as precise detection and identification of maize leaf azimuth and male ears [24,25]; accurate, objective, and efficient prediction of maize kernel breakage and kernel segregation [26,27]; achieving high-precision plant counting, row and plant spacing measurements, and missing plant detection [28]; and distinguishing embryonic and non-embryonic traits of maize kernels [29]. Therefore, further exploration is required to enhance the utilization of machine learning for assessing the drought tolerance of phenotypic traits during the maize seedling stage.

This study focused on 78 widely cultivated maize hybrid varieties in China. By integrating multi-phenotypic data with machine learning methods, it aims to analyze the effects of drought stress on maize seedling traits, identify key traits and optimal predictive models for assessing maize seedling drought tolerance, and evaluate the predictive performance of different machine learning methods. Ultimately, this analysis seeks to enhance the breeders’ and farmers’ understanding of the drought tolerance mechanisms during the maize seedling stage, facilitating the development of more efficient drought tolerance strategies and providing a theoretical foundation for the breeding and selection of maize varieties with enhanced drought resistance.

## 2. Materials and Methods

### 2.1. Test Materials

Seventy-eight maize hybrids were selected for the experiment (Appendix A, Table A1). These maize hybrids originate from different provinces of China and include varieties with large planting areas, sweet corn varieties, and glutinous corn varieties, with the aim of covering as many different types of maize varieties as possible.

### 2.2. Experimental Design

The experiment was conducted at the Experimental Station of the College of Agriculture of Tarim University, employing a potting method to induce drought stress. Each test sample consisted of 90 clean and uniform full seeds, which were sterilized using 75% alcohol, rinsed with distilled water, and subsequently dried on sterilized filter paper for further use. A nutrient soil blend combined with indigenous soil served as the growth substrate, with a nutrient bowl (35 cm high × 32 cm diameter) utilized as the growing container. The soil was evenly distributed, bagged, and thoroughly watered before planting. The weight of each bag was measured using an electronic scale to verify the uniformity of soil distribution, and subsequent weight measurements were taken after watering through the designated watering orifice. Six bags of each material were planted in three replicates and two treatment categories: normal watering (CK) and water stress (Drought). The next steps were as follows: sow 15 seeds per bag and retain 10 seedlings with consistent growth after germination; follow regular watering until the third leaf collar stage; and at the third leaf collar (V3) stage, water to field capacity. Seedlings were then subjected to water stress by withholding water for 10 days. Meanwhile, the normal watering group received 500 mL of water every 2 d to ensure adequate moisture. After 10 d of stress, 5 uniform and consistent seedlings were selected from each bag for the determination of eight phenotypic traits, including plant height, stem thickness, and chlorophyll content.

### 2.3. Item Determination

Five seedlings of each variety with uniform and even growth were selected for the determination of the indices, with results recorded to the nearest 0.01. Plant height was determined using a straightedge, extending from the stem base to the highest leaf blade [30]. Maximum stem thickness was determined at the cotyledons of the plant using a caliper gauge [30]. The root count was directly recorded as numerical values [31]. Chlorophyll SPAD values were measured at the widest section of fully expanded leaves utilizing a handheld portable chlorophyll meter, the model SPAD-502Plus, manufactured by Konica Minolta, Inc., based in Tokyo, Japan [32]. Following the desoiling, washing, and treatment of the seedlings, the aboveground and underground parts of the maize seedlings were separated with clean stainless-steel scissors, and then the surfaces of the aboveground and underground plants were rinsed and dried with deionized water. Aboveground and underground weights were determined using a precision electronic balance accurate to 1/1000th; the samples were segregated into kraft paper bags and subjected to heat treatment in a laboratory oven at 105 °C for 30 min, followed by drying at 70 °C to 80 °C until reaching a constant weight, and weighed again using a precision electronic balance accurate to 1/1000th as the pure aboveground weight and pure underground weight [33].

### 2.4. Data Processing

#### 2.4.1. Data Standardization

In this study, the data obtained for each trait index was pre-processed utilizing the z-score normalization method. Data normalization is implemented to standardize the scale differences, improve the efficiency of model training, facilitate the subsequent use of machine learning model training and analysis, and improve the stability and convergence speed of the model.

$$z_{i}=\frac{x_{i}-\mu }{\sigma }$$

where $x_{i}$ represents a feature value of the original dataset, *μ* denotes the mean (average) of the dataset, σ signifies the standard deviation of the dataset, and $z_{i}$ indicates the normalized value.

#### 2.4.2. Data Set Division

In this study, eight phenotypic traits’ data from 78 maize varieties were divided into a training dataset and a test dataset. Among them, 70% of the seedling characterization data were allocated for training to enable the model to grasp the patterns and trends within the seedling phenotypic characterization data. The remaining 30% of the seedling phenotypic characterization data were designated as a test set to evaluate the model’s capacity to generalize to unseen data. A 70% training set allows the model to learn enough features and patterns, improving its generalization ability. A 30% test set provides sufficient samples for objective model evaluation. To guarantee the representativeness of the training and test sets, random sampling was implemented during the partitioning process to prevent the model’s performance from being influenced by the data order. The training dataset serves as the sample dataset utilized for parameter learning and adjustment, while the test dataset is employed for assessing model performance post-training completion.

### 2.5. Model Selection and Evaluation Metrics

To comprehensively assess the effects of normal watering and 10 days of drought stress on eight phenotypic traits of maize seedlings, we first conducted a descriptive statistical analysis to understand the basic characteristics of each trait under different treatments. Subsequently, we performed an analysis of variance (ANOVA) to detect the significant effects of drought stress treatment on each phenotypic trait. Through descriptive statistical analysis and ANOVA, we were able to systematically evaluate the impact of drought stress on the phenotypic traits of maize seedlings and provide a solid data foundation for the subsequent machine learning model analysis.

In this study, to ascertain the significant features affecting drought tolerance in maize seedlings and ensure the accuracy of model predictions, we employed three machine learning methods using Python 3.12: random forest (RF) [34,35], K-nearest neighbors (KNN) [36], and eXtreme gradient boost (XGBoost, version Python 3.12) [37,38]. RF is an ensemble learning method based on multiple decision trees, which enhances the model’s stability and predictive power through voting. It introduces randomness on the basis of decision trees, making the model more generalizable and reducing the problem of overfitting. KNN is a supervised learning algorithm based on distance metrics and is commonly used for classification tasks. The core idea of KNN is to identify the K-nearest neighbors to a target sample based on distance measurements and then make predictions based on the majority class of these neighbors. XGBoost is an ensemble learning algorithm based on Gradient Boosted Decision Trees. It constructs multiple decision trees, with each tree attempting to correct the prediction errors of the previous one, thereby incrementally improving the overall model performance. XGBoost is known for its fast training speed, high model accuracy, and strong flexibility. The three models complement each other in terms of processing capabilities and application scenarios, allowing for a more comprehensive prediction of drought tolerance in maize seedlings.

In machine learning, feature importance refers to the magnitude of the role each feature plays in predicting the target variable; that is, it measures the contribution of each feature to the model’s prediction results. Feature contribution analysis focuses on explaining the contributions made by each feature in the model’s prediction results for individual samples. In this study, the identification of key features impacting drought tolerance in maize seedlings was conducted through feature importance (FI) ranking to enhance prediction efficiency and accuracy. Interpretation of model prediction results through feature contribution decomposition reveals the synergistic effect of individual features in seedling drought tolerance prediction, aiding in comprehending the model’s decision-making process. The performance of three different models is evaluated using standard evaluation metrics, given in Equations (1)–(8), respectively.

The Cross-Entropy (*CE*) loss function is a commonly used loss function for classification problems, especially suitable for binary classification tasks (such as drought-tolerant vs. non-drought-tolerant classification). It evaluates and quantifies the discrepancy between the model’s predicted values and the actual values. A smaller *CE* value indicates a closer alignment between the predicted and true values, indicating that the model performance is better. Additionally, when using the *CE* loss function, we can also assess the feature importance (loss: ce), which helps us understand the contribution of each feature to the model’s predictions. This provides further insights into which features are most relevant for the classification task. The formula is given by

(1) $$CE=-\frac{1}{n}\sum_{i=1}^{N}\left[y_{i}\log\left({\hat{y}}_{i}\right)+\left(1-y_{i}\right)\log\left(1-{\hat{y}}_{i}\right)\right]$$

where $y_{i}$ represents the true value of the ith sample, and ${\hat{y}}_{i}$ denotes the predicted probability of the ith sample.

*ACC* (accuracy) is a metric that indicates the proportion of samples correctly classified by the model. A value closer to 1 signifies superior classification performance of the model. The formula is given by

(2) $$ACC=\frac{TP+TN}{TP+TN+FP+FN}$$

where *TP* (True Positive) denotes the count of varieties correctly predicted by the model to be drought tolerant, *FN* (False Negative) represents the count of varieties incorrectly predicted by the model to be non-drought tolerant, *TN* (True Negative) signifies the count of varieties correctly predicted by the model to be non-drought tolerant, and *FP* (False Positive) indicates the count of varieties incorrectly predicted by the model as drought tolerant.

The *R*^2^ assesses the degree to which the fitted model accounts for the variance in the observed data, with values ranging from 0 to 1. Higher values of R^2^ indicate a more favorable fit of the model. The formula is given by

(3) $$R^{2}=1-\frac{\sum_{i=1}^{n}{\left(y_{i}-{\hat{y}}_{i}\right)}^{2}}{\sum_{i=1}^{n}{\left(y_{i}-{\bar{y}}_{i}\right)}^{2}}$$

where $y_{i}$ represents the true observation, $\bar{y}$ is the mean of the true observation, and ${\hat{y}}_{i}$ denotes the predicted value.

*RMSE* (Root Mean Square Error) is the square root of the error between the predicted value and the true value, serving as a measure of the average error between the fitted curve and the actual observed data. A smaller *RMSE* value indicates that the fitted model better fits the actual observed data. The *RMSE* formula is given by

(4) $$RMSE=\sqrt{\frac{1}{n}\sum_{i=1}^{n}{\left(x_{i}-y_{i}\right)}^{2}}$$

where $x_{i}$ represents the predicted value of the ith sample and *y_i_* denotes the true value of the ith sample.

*Sensitivity* (true positive rate, *TPR*) indicates the proportion of drought-tolerant varieties correctly identified by the model. A higher sensitivity value signifies the model’s proficiency in identifying genuine drought-tolerant varieties. It is defined as

(5) $$\mathrm{Sensitivity}=\mathrm{True}\;\mathrm{Positive}\;\mathrm{Rate}\;(\mathrm{TPR})=\frac{TP}{TP+FN}$$

*Specificity* (true negative rate, *TNR*) indicates the ratio of non-drought-tolerant varieties correctly classified by the model. Higher specificity values indicate the model’s effectiveness in recognizing genuine non-drought-tolerant varieties. It is defined as

(6) $$Specificity=True\;Negative\;Rate\;\left(TNR\right)=\frac{TN}{TN+FP}$$

The false positive rate (*FPR*) is defined as the proportion of actual negative samples that are incorrectly predicted as positive. It is the complement of specificity and is calculated as

(7) $$FPR=1-specificity=\frac{FP}{FP+TN}$$

The *ROC* curve is a series of different true positive rates (*TPR*) and false positive rates (*FPR*) calculated by varying the categorization thresholds and plotting them as a curve. The area below the curve is the *AUC* value. The *AUC* value ranges from 0 to 1; a higher *AUC* value indicates superior classification performance of the model. The *AUC* is computed as

(8) $$AUC=\int_{0}^{1}TPR\left(f\right)df$$

$TPR\left(f\right)$ is the true positive rate corresponding to a false positive rate (FPR) of f.

*AUC* loss refers to the decrease in the *AUC* value after permuting a feature, that is, the original *AUC* value minus the *AUC* value after permutation. By calculating “1 − *AUC* loss,” the importance of features can be normalized to the interval 0 to 1, which facilitates the comparison of the importance of different features. The closer the value is to 1, the more important the feature is.

## 3. Results

### 3.1. Descriptive Statistical Analysis of Seedling Phenotypic Traits Under Drought Stress

**Figure 1:**
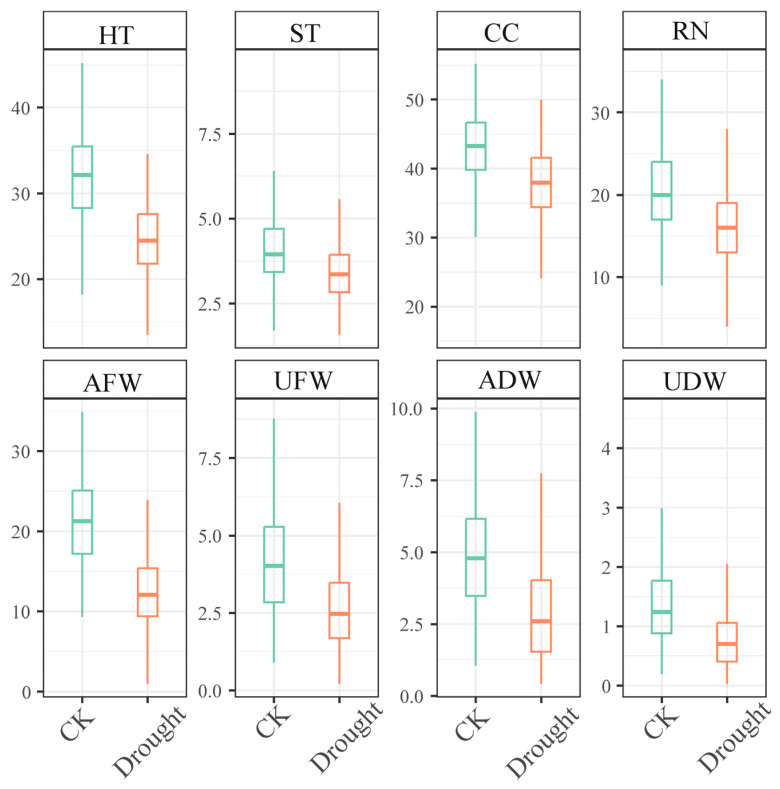
The box line plots display the data distribution for normal watering (CK) and water stress by withholding water for 10 days (Drought). Abbreviations used in the figure are defined as follows: HT: height, ST: stem, CC: chlorophyll content, RN: root number, AFW: aboveground fresh weight, UFW: underground fresh weight, ADW: aboveground dry weight, UDW: underground dry weight.

To initially understand the differences in the eight phenotypic traits of maize under normal watering and drought stress conditions, it is necessary to conduct a descriptive statistical analysis of these traits. As illustrated in Figure 1 and Appendix A, Table A2, drought stress significantly inhibited the morphogenesis and material accumulation in maize seedlings. Compared with the control, drought stress significantly reduced the plant height and aboveground fresh weight of maize seedlings while exerting a lesser impact on root number. The coefficients of variation of plant height and stem thickness decreased, whereas those for other parameters increased. This trend suggests that under drought conditions, maize plants experienced constrained water availability, hindering their growth. This constraint was observed across different individuals, leading to a general limitation in the variations in plant height and stem thickness and diminishing inter-individual disparities. Variations in physiological metabolism among individual plants, including differences in root system water uptake capacity, leaf photosynthesis, hormone regulation mechanisms, and other factors, contribute to distinct responses. When subjected to drought stress, these divergences result in varying trends in chlorophyll content, root number, fresh weight, and dry weight among plants, consequently elevating the coefficient of variation.

**Figure A1:**
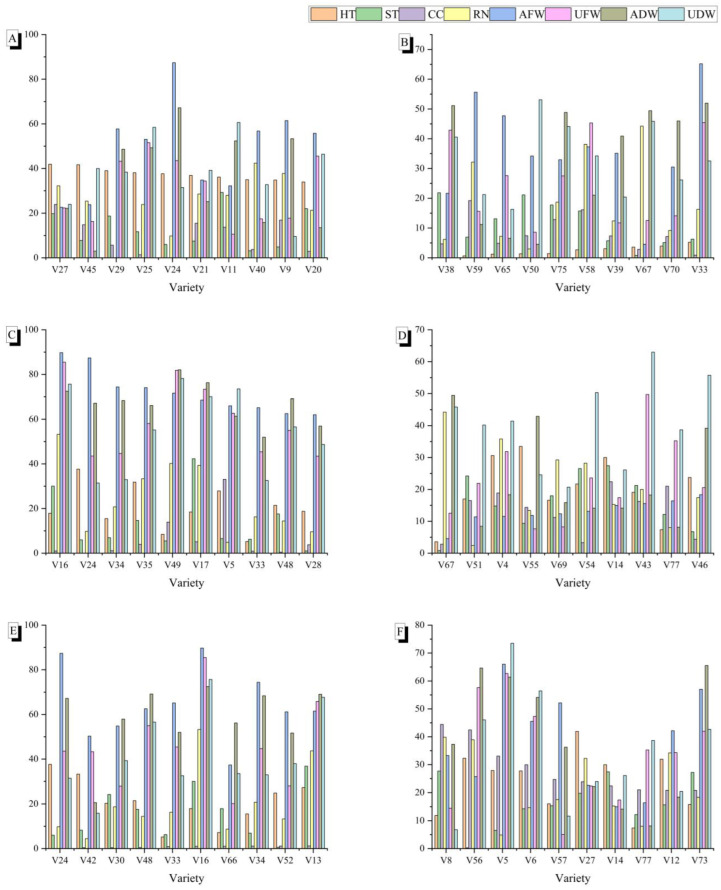
Drought tolerance ranking of maize varieties based on key growth indices. Note: Abbreviations used in the figure are defined as follows: HT: height, ST: stem, CC: chlorophyll content, RN: root number, AFW: aboveground fresh weight, UFW: underground fresh weight, ADW: aboveground dry weight, UDW: underground dry weight. Ten representative varieties showed maximum (**A**) and minimum (**B**) percentage reduction in HT, maximum (**C**) and minimum (**D**) percentage reduction in UP, and minimum (**E**) and maximum (**F**) percentage reduction in CC. The horizontal axis shows variety number, and the vertical axis shows range of effects of 10-day no-watering treatment on eight phenotypic traits.

Drought stress significantly impedes maize seedling growth and induces measurable phenotypic alterations; among them, plant height, aboveground fresh weight, and chlorophyll content are the key indicators for distinguishing whether a maize seedling is drought tolerant or not. Therefore, we ranked the drought tolerance of 78 maize varieties based on the magnitude of changes in the effects of 10-day drought treatments on the above three indexes and demonstrated 10 representative varieties whose plant height, aboveground fresh weight, and chlorophyll content were most and least affected by drought stress, and the results are shown in Appendix A, Figure A1. This low-cost, rapid, and simple method to distinguish seedlings for drought tolerance is important for the early screening of drought-tolerant resources by breeders.

### 3.2. Analysis of Variance (ANOVA) of Seedling Phenotypic Traits Under Drought Stress

To determine whether drought stress has a significant impact on the phenotypic traits of maize, we conducted an analysis of variance (ANOVA). As shown in Table 1, the results of the analysis of variance revealed a notably significant impact of drought stress on the eight assessed traits. Among them, the drought treatment had the greatest effect on plant height, followed by the effect on aboveground fresh weight. The interactions between varieties and drought were larger for plant height, aboveground fresh weight, and aboveground dry weight, indicating that there were differences in the response to drought among varieties, with some varieties showing greater tolerance or sensitivity, while the interactions for stem and root number were not significant, indicating that the response of these indicators to drought converged among varieties.

### 3.3. Feature Importance Analysis of Phenotypic Traits at Seedling Stage

**Figure 2:**
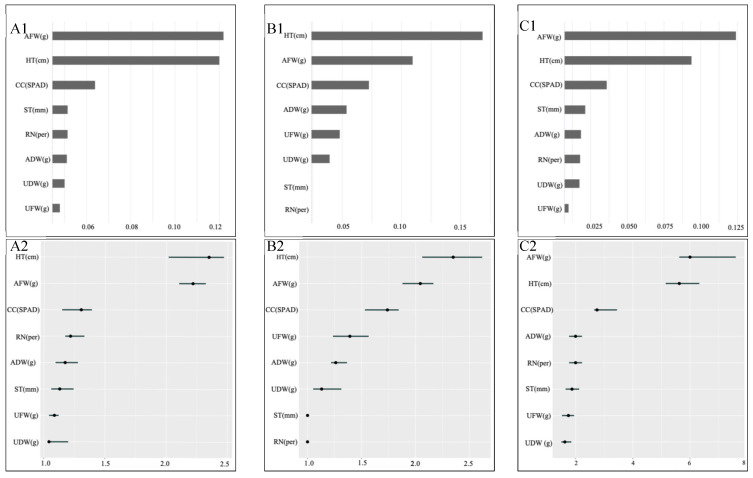
Feature importance of the three models based on two methods. (**A1**) (RF), (**B1**) (KNN), and (**C1**) (XGBoost) are based on 1 − *AUC* loss after permutations and (**A2**) (RF), (**B2**) (KNN), and (**C2**) (XGBoost) are based on feature importance (loss: ce). The vertical axes represent the different traits, i.e., the 8 abbreviations of the listed traits, including HT: height, ST: stem, CC: chlorophyll content, RN: root number, AFW: aboveground fresh weight, UFW: underground fresh weight, ADW: aboveground dry weight, UDW: underground dry weight. The horizontal axes represent the degree to which each feature contributes to the predictive capability of the model. Numbers indicate value ranges, reflecting feature importance for predictive ability. A higher value indicates a more substantial contribution to the drought tolerance classification model.

Feature importance is a measure of how much each feature contributes to the model’s predictions, and it reflects how much a feature influences the predictions in the model. To identify the key features influencing maize seedling drought tolerance, we conducted a feature importance analysis on the three models used in this study. Thus, we were able to identify which phenotypic traits have the most critical impact on maize seedling drought tolerance. In this study, the assessment of feature importance primarily employs two methods, as depicted in Figure 2. One method is centered on the “One minus *AUC* loss after permutations” approach, while the other is based on the “Feature Importance (loss: ce)” method. These two methods assess the contributions of features to model predictive ability from different perspectives. However, they differ in their evaluation methods and focal points. The “One minus *AUC* loss after permutations” method focuses more on the stability of model classification performance due to feature permutations, whereas the “Feature Importance (loss: ce)” method emphasizes the direct impact of features on model prediction accuracy. By combining these two methods, we can gain a more comprehensive understanding of how features influence the model, thereby optimizing the model and feature selection.

We used the first method to rank all features based on the “1 − *AUC* loss” value to identify the key features that contribute the most to predicting drought tolerance in the early growth stage of corn. Through this approach, we were able to quantify the contribution of each feature to model performance and identify the most influential phenotypic traits for drought tolerance prediction. Figure 2A1–C1 represent the feature importance ranking for each model, with the importance values of individual features arranged in descending order via the blue bar graph. This graphical representation facilitates the quick identification of the most crucial features. The outcomes highlight that features such as aboveground fresh weight, plant height, and chlorophyll content hold significant importance, underscoring their pivotal role in model prediction. The second method is based on feature importance (loss: ce). Figure 2A2–C2 show the feature importance values ranked from high to low by this method, with dots indicating the importance of each feature and accompanying confidence intervals, emphasizing the stability and reliability of feature importance. The findings reveal that plant height, aboveground fresh weight, and chlorophyll content remain prominent features with high importance, although the sequence of features varies slightly across different models. A greater feature importance value signifies a more substantial contribution to the predictive capacity of the model. Among the three machine learning models, both feature importance ranking methods consistently highlight plant height, aboveground fresh weight, and chlorophyll content as the fundamental features influencing predictive performance. Among these features, the stability of chlorophyll content’s ranking is notable, consistently securing the third position across various models and methods. Conversely, the relative importance of plant height and aboveground fresh weight changes dynamically, with their ranking positions varying slightly based on model disparities and analytical methodologies. Notably, in the KNN model, the significance of aboveground dry weight and underground fresh weight surpasses that of other models, ranking relatively higher in terms of feature importance. In contrast, the importance of stem is relatively lower, albeit it still exerts some influence in the RF and XGBoost models. Overall, the three models consistently identify plant height, aboveground fresh weight, and chlorophyll content as pivotal indicators for predicting drought tolerance, underscoring their paramount importance for maize seedling drought tolerance.

### 3.4. Decomposition of Feature Contributions for Seedling Phenotypic Traits

**Figure 3:**
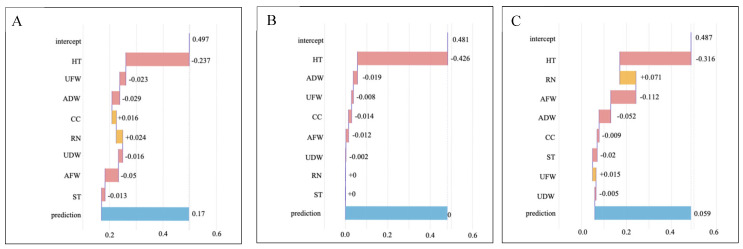
Feature contribution of three models: RF (**A**), KNN (**B**), and XGBoost (**C**). The horizontal axes represent the cumulative contribution to the model’s predicted values. The numbers indicate value ranges, starting from a base value and accumulating to the final predicted value through each feature’s contribution. The vertical axes show feature abbreviations, representing the different features used in the model. Bars of different colors show positive and negative directions of feature contribution, with red representing positive and blue representing negative contributions. Displayed numerical values indicate the magnitude of contributions.

To further quantify the contribution of each phenotypic trait to the model’s prediction of drought tolerance, we conducted an analysis of the feature contribution rate. This allowed us to clarify the relative contributions of each phenotypic trait to the model’s prediction of drought tolerance. Feature contribution analysis explains the contribution made by each feature in the model’s prediction results for individual samples. Apart from assessing feature importance in models, interpreting the contribution values of individual features to prediction results is a critical aspect of model application. This process aids in comprehending the model’s decision-making rationale for specific samples and delineates how each feature impacts particular predictions. Figure 3A–C show the breakdown plots for the three models. The intercept represents the baseline prediction of the model in the absence of feature influences, while the prediction denotes the final output derived from the cumulative contributions of all features. The contribution of each feature (whether positive or negative) is cumulatively aggregated to generate the prediction.

Consistent across all three models, plant height was consistently identified as exerting a notable negative impact, indicating that excessive plant height detrimentally affects drought tolerance—a shared observation among the models. In the RF model (A), the features chlorophyll content and root number showed positive contributions, while in the XGBoost model (C), RN and underground fresh weight exhibited positive effects. Notably, the KNN model failed to capture features with positive contributions. The features underground fresh weight, aboveground dry weight, underground fresh weight, and underground dry weight had different contributions in the different models, predominantly manifesting a negative influence on predictions in most instances.

### 3.5. Comparison of Model Training and Testing Results

The true value of a machine-learning model lies in improving the generalization ability for unseen test-set data by learning the features and patterns of training-set data, thereby validating the performance and reliability of the model. To evaluate the performance of the three models in predicting maize seedling drought tolerance, we trained and tested each model. As depicted in Table 2, following model training, the chlorophyll content, *ACC*, and *AUC* values of the RF model and the KNN model exhibit close proximity between the training and test sets. This similarity suggests the absence of evident overfitting in the model and signifies its robust generalization capability, particularly in effectively discerning drought-tolerant maize. The *CE* values of the XGBoost model in the training set and the test set are notably low, with the test set loss being even lower than that of the training set. This disparity indicates superior model performance in the test set and underscores the model’s adeptness in comprehensively learning complex features during training without succumbing to overfitting. The *ACC* values exhibit notably high levels in both the training and test sets, with the accuracy in the test set surpassing that of the training set. This disparity further underscores the model’s strong generalization capacity and stability. Similarly, the *AUC* values demonstrate high performance levels in both the training and test sets, indicating the XGBoost model’s proficiency in effectively discriminating between positive and negative samples.

*R*^2^ and *RMSE* are commonly utilized metrics to assess the predictive ability and accuracy of models. Table 3 illustrates that the RF model and KNN model exhibit comparable *R*^2^ and *RMSE* values across both the training and test sets, indicating the absence of overfitting. The values, which show that these two models demonstrate a lower *R*^2^ (0.606) and higher *RMSE* (0.314) in the test set, suggest limited predictive capabilities of these models. The XGBoost model demonstrates a higher *R*^2^ (0.863) in the test set compared to the training set (0.809), potentially attributed to the regularization parameter effectively mitigating overfitting and enhancing generalization. Meanwhile, the remarkably low *RMSE* values (0.218 for the training set and 0.185 for the test set) signify minimal error between the predicted and true values. This indicates the model’s exceptional performance on training data and its effective generalization to new data, rendering it highly practical.

### 3.6. Performance Evaluation of Machine Learning Models for Seedling Phenotypic Traits

Assessing the holistic performance of a learning model is crucial in gauging its practical applicability. To select the most suitable model for assessing maize seedling drought tolerance, we conducted a comparative analysis of the performance of the three models used in this study. We evaluated their performance in predicting maize seedling drought tolerance and determined which model had the best predictive performance. We employ the *ROC* curve and the corresponding *AUC* value as primary evaluation metrics to comprehensively evaluate the performance of the three models on the test dataset.

**Figure 4:**
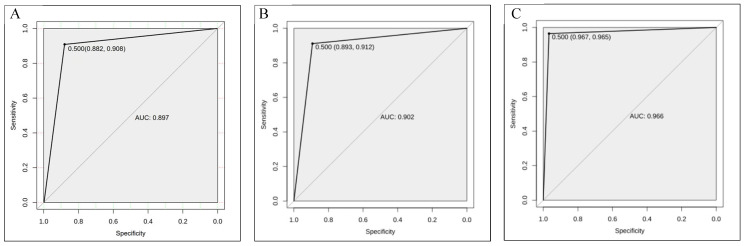
The *ROC* curves for three different models: RF (**A**), KNN (**B**), and XGBoost (**C**). The horizontal axes represent specificity, which is the proportion of samples in the negative category (i.e., samples that are actually in the negative category and are predicted to be in the negative category) that are correctly identified by the model. The vertical axes represent sensitivity, i.e., TPR, the proportion of samples in the positive category (i.e., samples that are actually in the positive category and are predicted to be in the positive category) that the model correctly identifies.

The *ROC* curves of the three machine learning models are presented in Figure 4. The *ROC* curve is a prevalent tool for evaluating the performance of classification models, illustrating the classification efficacy under various thresholds by plotting the relationship between the true positive rate (sensitivity) and the false positive rate (1 − specificity). A high sensitivity aids in correctly identifying drought-tolerant varieties, while a high specificity indicates that varieties designated as drought-tolerant are predominantly accurate. For the dichotomous problem of distinguishing whether corn is drought-tolerant or not, 0.5 is a commonly used threshold value: when the probability is greater than 0.5, it indicates that maize is more likely to be drought-tolerant in the seedling stage; on the contrary, it is more likely to be not drought-tolerant when the probability is less than 0.5. Both the RF and KNN models have lower *ROC* curves and *AUC* values than the XGBoost model. In the XGBoost model, the sensitivity was 0.967, the specificity was 0.965, and the *AUC* value was 0.966. All three models achieved *AUC* values exceeding 0.9, signifying excellent performance in discriminating between the drought-tolerant and non-drought-tolerant varieties of maize at the seedling stage. Notably, the XGBoost model exhibited the highest *AUC* value, indicating its superior performance in distinguishing the drought tolerance status of maize plants.

## 4. Discussion

### 4.1. Changes in Phenotypic Traits of Maize Seedlings Under Drought Stress

Drought stress is a prevalent environmental challenge in agricultural production, exerting a profound impact on crop growth, development, and particularly yield. Investigating the phenotypic changes in maize seedlings under drought stress is crucial for the breeding and selection of drought-tolerant varieties. Under drought stress, the root system encounters challenges in water absorption from the soil due to decreased soil moisture levels, triggering alterations in root morphology and structure that subsequently impact aboveground growth. In this study, drought stress treatments led to a notable reduction in the fresh weight of the root system compared to normal irrigation conditions. However, the impact on dry weight was not significant, suggesting that drought primarily affected the water content and growth of the root system directly, while exerting a lesser influence on root structure or dry matter accumulation. In addition, the number of roots was also affected by drought stress [39]. The coefficients of variation for underground dry weight and aboveground dry weight were significantly higher than those of the control, indicating that drought exacerbated phenotypic differences among plants [40]. Among the measured traits, the aboveground fresh weight was most significantly affected by drought stress, indicating that aboveground fresh weight was more sensitive to drought [41], as evidenced by lower plant height, thinner stem thickness, and lower aboveground fresh weight. The decrease in chlorophyll content is attributed to drought-induced closure of leaf stomata, which restricts carbon dioxide entry, consequently constraining the dark phase of photosynthesis. Decreased photosynthetic efficiency triggers photoinhibition, leading to damage to photosystem II (PS II), thereby accelerating chlorophyll degradation [42]. This process culminates in diminished aboveground biomass accumulation in maize seedlings.

### 4.2. Analysis of the Importance and Contribution of Phenotypic Traits of Maize Seedlings Under Drought Stress

Key traits related to drought tolerance in maize seedlings are crucial for understanding drought tolerance mechanisms and guiding agricultural production. Through trait importance analysis, we identify the phenotypic traits that contribute most to drought tolerance. In this study, three machine learning models were developed to predict maize drought tolerance based on eight phenotypic traits measured during the seedling stage. Three machine learning models collectively revealed that seedling-stage plant height, aboveground fresh weight, and chlorophyll content have a critical influence on model predictions: the high importance scores of these features in the model indicate that they contribute the most to the prediction of drought tolerance. The analysis of feature contribution rates further indicates that plant height and aboveground fresh weight account for a larger proportion of the model’s predictions. This further validates their importance in the assessment of drought tolerance. Among these traits, plant height was identified as the most consistently influential factor affecting maize seedlings’ drought tolerance. This indicates that plants with higher seedling heights tend to have poorer drought tolerance. Excessively tall plants or excessive aboveground biomass can lead to increased transpiration, thereby reducing their drought tolerance. This is related to increased water transport efficiency and transpiration water consumption, as well as reduced photosynthetic efficiency [43,44]. The root system serves as the primary organ for soil water absorption in plants, and under drought stress, a large number of roots or a well-developed root system can enhance the water absorption capacity of the root system in arid environments, consequently bolstering the drought tolerance indirectly [45]; root systems will respond to drought stress by reshaping their structure and altering their material content [46,47,48]. The variations in maize leaf chlorophyll content affect its photosynthetic efficiency [49,50], subsequently influencing its water-use efficiency, resilience in arid settings, and drought tolerance [51].

### 4.3. Performance of Machine Learning Models for Drought Tolerance Prediction in Maize Seedlings

Machine learning models have significant advantages in processing complex data and performing predictive tasks. By comparing the performance of different models, we can select the most suitable model to assess the drought tolerance of maize seedlings, thereby providing technical support for the selection and breeding of drought-tolerant varieties. The evaluation results of the three machine learning methods on the training and test sets revealed superior performance by the XGBoost model, exhibiting a higher *R*^2^ value (0.863) in classification tasks. This indicates the model’s proficient ability to elucidate variations in drought tolerance at the seedling stage of maize. The lower *RMSE* value (0.185) indicated close proximity between the predicted drought tolerance scores and the actual values, reflecting enhanced prediction accuracy. In the model performance evaluation, the XGBoost model outperformed both the RF model and the KNN model in the *ROC* curve at the 0.5 threshold. The higher *AUC* value (0.966) indicates its strong utility in classifying maize drought tolerance data, offering dependable predictive assistance for practical decision-making processes. Although the RF and KNN models demonstrated superior stability (training and test set differences < 2%), they exhibited a limited predictive ceiling (*R*^2^ ≈ 0.6). This limitation could stem from the models’ challenges in capturing high-dimensional data and non-linear associations, potentially mismatching the intricate characterization of maize drought tolerance interactions. The variations in model performance also underscore the multiscale regulatory intricacies inherent in maize drought tolerance [51].

## 5. Conclusions

We collected data on the eight phenotypes of 78 maize hybrids at the seedling stage under normal watering and 10-day water-withholding treatments and conducted a comprehensive analysis of drought-stressed maize seedlings utilizing three machine learning models—evaluating feature importance, feature contribution decomposition, and predictive performance. In the evaluation of model performance, the XGBoost model, with a higher R^2^ value of 0.863, a higher AUC value of 0.966, and a lower RMSE value of 0.185, shows strong performance. It also demonstrates superior predictive stability and interpretability compared to the RF and KNN algorithms. Machine learning effectively identifies drought-responsive phenotypic signatures, namely plant height, aboveground weight, and chlorophyll content, as three key phenotypic indicators to distinguish the maize varieties as drought tolerant or not. Based on the three key phenotypic indicators identified above, we recommend prioritizing their stability validation in subsequent field trials. The assessment criteria established through this process will provide a cost-effective, rapid, and user-friendly technical framework to support early drought warning and systematic screening of drought-resilient germplasm resources.

Although this study provides a novel method for evaluating drought tolerance at the maize seedling stage, several limitations remain. First, while the 78 hybrid varieties encompass genetic diversity, the variation in the inherent genetic backgrounds among different varieties was not accounted for. Future studies could focus on specific germplasm types to refine this approach. Second, the current research included only eight easily quantifiable phenotypic traits, omitting critical traits such as three-dimensional root architectural parameters (e.g., root volume, branching patterns) and dynamic physiological indicators (e.g., stomatal conductance, photosynthetic rate). Future work should integrate high-precision root scanners and real-time monitoring of leaf photosynthesis to construct multidimensional phenotype–environment interaction datasets, thereby enhancing the model’s capacity to decipher complex drought-resistance mechanisms. To address these gaps and enhance predictive capabilities, future research could integrate machine learning models with drone-based image recognition technology to enable comprehensive seedling condition monitoring throughout the growth cycle, thereby establishing disaster assessment frameworks and yielding prediction systems. Through the fusion of multi-source datasets, this approach will facilitate the translation of machine learning into practical applications in crop stress-resilient breeding and precision monitoring.

## Figures and Tables

**Table 1:** Analysis of variance (ANOVA) for phenotypic traits of maize under drought stress during the seedling stage.

Source of Variation	Index	Sum of Squares	Mean Square	F Value
Variety	HT	7950.86	103.26	11.40 **
	ST	232.28	3.02	4.06 **
	CC	6642.84	86.27	5.57 **
	RN	7348.14	95.43	5.40 **
	AFW	10,801.85	140.28	15.51 **
	UFW	1255.07	16.30	20.88 **
	ADW	1474.50	19.15	18.81 **
	UDW	151.63	1.97	7.70 **
Drought	HT	9577.41	9577.41	1056.99 **
	ST	89.80	89.80	120.96 **
	CC	4995.23	4995.23	322.47 **
	RN	3935.26	3935.26	222.72 **
	AFW	15,514.73	15,514.73	1715.35 **
	UFW	403.69	403.69	517.05 **
	ADW	651.10	651.10	639.59 **
	UDW	68.26	68.26	267.04 **
Variety * Drought	HT	3356.49	43.59	4.81 **
	ST	52.88	0.69	0.93
	CC	3416.26	44.37	2.86 **
	RN	1581.54	20.54	1.16
	AFW	3911.40	50.80	5.62 **
	UFW	189.91	2.47	3.16 **
	ADW	319.69	4.15	4.08 **
	UDW	33.41	0.43	1.70 **
Error	HT	5654.07	9.06	
	ST	463.26	0.74	
	CC	9665.98	15.49	
	RN	11,025.60	17.67	
	AFW	5643.86	9.04	
	UFW	487.19	0.78	
	ADW	635.22	1.02	
	UDW	159.50	0.26	

Note: * indicates significant correlation (*p* < 0.05); ** indicates highly significant correlation (*p* < 0.01). Abbreviations used in the table are defined as follows: HT: height, ST: stem, CC: chlorophyll content, RN: root number, AFW: aboveground fresh weight, UFW: underground fresh weight, ADW: aboveground dry weight, UDW: underground dry weight.

**Table 2:** Performance comparison of machine learning models.

Model	*CE*		*ACC*		*AUC*	
	Train	Test	Train	Test	Train	Test
RF	0.090	0.098	0.910	0.902	0.963	0.955
KNN	0.090	0.098	0.910	0.902	0.975	0.976
XGBoost	0.048	0.034	0.952	0.966	0.994	0.993

Notes: Smaller *CE* value indicates a closer alignment between the predicted and true values, reflecting improved model performance. *ACC* is a metric that indicates the proportion of samples correctly classified by the model. A value closer to 1 signifies superior classification performance of the model. A higher *AUC* value indicates superior classification performance of the model.

**Table 3:** *R*^2^ and *RMSE* of RF, KNN, and XGBoost in training and test sets.

Model	*R* ^2^		*RMSE*	
	Train	Test	Train	Test
RF	0.641	0.606	0.300	0.314
KNN	0.641	0.606	0.300	0.314
XGBoost	0.809	0.863	0.218	0.185

Notes: Higher values of *R*^2^ indicate a more favorable fit of the model. A smaller *RMSE* value indicates that the fitted model better fits the actual observed data.

**Figure A2:**
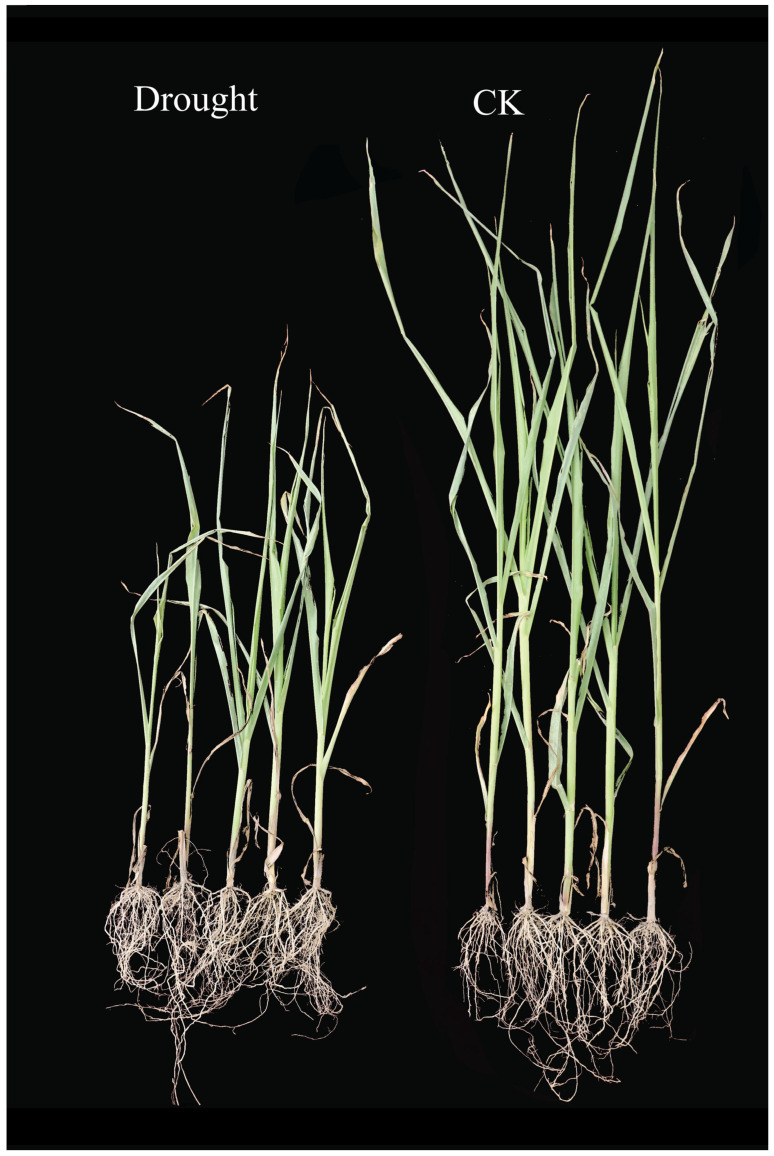
Drought impact on maize seedlings: drought (water stress by withholding water for 10 days) VS CK (normal watering).

## Data Availability

The original contributions presented in this study are included in the article; further inquiries can be directed to the corresponding author.

## Abbreviations

The following abbreviations are used in this manuscript:

ML	Machine learning
RF	Random Forest
KNN	K-Nearest Neighbors
XGBoost	Extreme Gradient Boosting
ROC	Receiver Operating Characteristic
AUC	Area Under the Curve
CE	Cross-Entropy Loss
ACC	Accuracy
R^2^	Coefficient of Determination
RMSE	Root Mean Square Error
Min	Minimum
Max	Maximum
Ave	Average
CV	Coefficient of Variation
SD	Mean Square
HT	Height
ST	Stem
CC	Chlorophyll Content
RN	Root Number
AFW	Aboveground Fresh Weight
UFW	Underground Fresh Weight
ADW	Aboveground Dry Weight
UDW	Underground Dry Weight

## Appendix A

**Table A1:** Seventy-eight maize hybrids that were selected for the experiment.

No.	Variety	No.	Variety	No.	Variety
V1	WoFeng188	V27	HuangJinnuo	V53	HuangNian5
V2	YouQi909	V28	BaiTiannuo7	V54	YuZhuxiangtiannuo
V3	XiMeng208	V29	FengNuo2	V55	XiMeng668
V4	XiMeng3358	V30	CaiNuo999	V56	LinYu1339
V5	HongXing528	V31	BaiNuo68	V57	HuXin338
V6	JiXing218	V32	HuangNuo688	V58	HuiTiannuo1
V7	JinFengjie	V33	ZengYu157	V59	JingZinuo218
V8	JiNongyu309	V34	XingNong1	V60	NongKeyu318
V9	JinAi588	V35	FengTian14	V61	JingCaitiannuo
V10	NongFu99	V36	BaiNuo1	V62	XinNuoyu10
V11	ZhongXing618	V37	CaiTiannuo118	V63	CaiTiannuo218
V12	HongXing990	V38	ZaoBaitianuo1	V64	TianJianuo1
V13	QiangZaocaitianno	V39	CaiTiannuo856	V65	FengNongcaitiannuo
V14	NuoYu2	V40	YuShuai3000	V66	SanMeng9599
V15	JingNuo2000	V41	HuangNuo518	V67	YuanYuan1
V16	HeiBao	V42	JingKenuo2000	V68	XuanHe8
V17	TianNuo828	V43	YuZhu3000	V69	QunCe888
V18	TianJianuo518	V44	XiangHe9918	V70	YuHe536
V19	ZiXiangnuo	V45	FuYu109	V71	XiMeng6
V20	CaiTiannuo8	V46	XianYu335	V72	BiXiang809
V21	PingAn1523	V47	GanTiannuo3	V73	HuXi712
V22	HengYu369	V48	KeNuo167	V74	WoFeng9
V23	XinNong008	V49	KeNuo2000	V75	XinYu66
V24	WuGu568	V50	BaoNuo5	V76	XinYu24
V25	TianXiangnuo9	V51	GanTiannuo2	V77	GanXin2818
V26	BaiFumei66	V52	ZaoNuo8	V78	XinYu81

**Table A2:** Descriptive statistics for phenotypic traits of maize under drought stress during the seedling stage.

Index	Min		Max		Ave		SD		CV (%)	
	CK	Drought	CK	Drought	CK	Drought	CK	Drought	CK	Drought
HT (cm)	21.18	20.12	39.34	30.36	31.81	24.8	4.48	3.04	14.09	12.27
ST (mm)	2.61	2	6.72	4.74	4.13	3.45	0.69	0.52	16.68	14.98
CC (SPAD)	33.6	25.94	49.32	47.84	43.03	37.97	3.30	3.91	7.66	10.29
RN (per)	12.2	7.4	27.4	24.2	20.59	16.1	3.50	3.31	17.01	20.53
AFW (g)	12.04	1.71	29.82	19.57	21.16	12.24	4.60	4.13	21.74	33.76
UFW (g)	1.34	0.33	7.58	7.07	4.15	2.71	1.44	1.29	34.84	47.65
ADW (g)	1.86	0.61	7.94	7.75	4.8	2.97	1.50	1.55	31.31	52.13
UDW (g)	0.39	0.18	2.71	2.32	1.39	0.8	0.57	0.40	40.58	49.92

Note: Abbreviations used in the table are defined as follows (the same applies hereafter): Min: minimum, Max: maximum, Ave: average, CV: coefficient of variation, HT: height, ST: stem, CC: chlorophyll content, RN: root number, AFW: aboveground fresh weight, UFW: underground fresh weight, ADW: aboveground dry weight, UDW: underground dry weight.
